# Hazardous Waste Disposal Enterprise Selection in China Using Hesitant Fuzzy PROMETHEE

**DOI:** 10.3390/ijerph17124309

**Published:** 2020-06-16

**Authors:** Xuedong Liang, Jinrui Miao, Yanjie Li, Xu Yang, Zhi Li

**Affiliations:** 1 The Economy and Enterprise Development Institute, Sichuan University, Chengdu 610065, China; liangxuedong@scu.edu.cn; 2 Business School, Sichuan University, Chengdu 610065, China; 2018225025102@stu.scu.edu.cn (J.M.); liyanjie@stu.scu.edu.cn (Y.L.); yangxu3671@163.com (X.Y.)

**Keywords:** hazardous waste, hazardous waste disposal enterprises, hazardous waste disposal enterprise evaluation index system, hesitant fuzzy linguistic glossary, PROMETHEE

## Abstract

Because of the urgent need to protect the environment, it has become vital to deal with the dangers and particularities associated with the growth in hazardous industrial waste. Governments have begun to expand their investments in the environmental protection industry and have tightened enterprise environmental management requirements. The 13th Five-Year Plan period in China, in particular, increased the focus on the environmental supervision and enterprise environmental management. Because of the specific qualities of many types of hazardous waste, most enterprises do not have the ability to process hazardous waste and therefore must outsource the disposal to third-party contractors. However, choosing suitable hazardous waste disposal enterprises (HWDE) can be difficult. Therefore, to assist in the selection of appropriate hazardous waste disposal enterprises, this paper developed a comprehensive evaluation index system for hazardous waste disposal enterprises (EISHWDE). As multi-criteria decision-making problems involve qualitative evaluations that have semantic ambiguity, hesitant fuzzy linguistic term sets (HFLTS) were introduced to increase the accuracy of the evaluation process, an analytic hierarchy process (AHP) used to determine the objective indicator weights, and PROMETHEE (Preference Ranking Organization Method for Enrichment Evaluation) employed to determine the final order for the selected enterprises. This research developed a scientific evaluation model that industrial waste enterprises (IWE) and related organizations could use to objectively and systematically select suitable hazardous waste disposal enterprises. Then, the problems of uncertainty and fuzzy semantics in the evaluation process were solved, and the weight of each selection criteria and the ranking of alternative enterprises are given.

## 1. Introduction

The global annual hazardous waste output from industrial sources was 3.3 × 10^8^ tonnes in 2011 [1]. If hazardous waste is not dealt with properly, there can be potential long-term and catastrophic effects on the water, the atmosphere, the soil and the environment. Therefore, hazardous waste environmental management, which involves solid waste generation, collection, storage, transportation, utilization and disposal, is an indispensable part of environmental pollution prevention and control.

The “China Statistical Yearbook” reported that the annual hazardous industrial waste in China increased from 9.52 million tonnes in 2001 to 69.37 million tonnes in 2017, an increase of 628.68%, and an average annual growth rate of 36.98%. Therefore, because of this massive rise in hazardous waste, the Chinese government has been encouraging greater investment in the environmental protection industry. The total social environmental protection investment during the 13th Five-Year Plan period will exceed CNY 17 trillion (USD 2.4 trillion). Because of these continuous efforts to improve environmental supervision on all levels, the environmental management requirements in companies and industries have become more standardized.

Consequently, hazardous waste management has become a key problem for enterprise environmental protection. While dealing with hazardous waste has become increasingly important, because China’s hazardous waste industry is little more than 20 years old, there remain many problems to be solved.

The “National Hazardous Waste List” of China divides hazardous waste into 50 categories; HW01-HW03 are related to medical waste and HW04-HW50 are all related to industrial waste from industrial production. At present, there are three major hazardous waste classification systems in the world, including the “Basel Convention”, the “European Waste Catalogue” and the “Code of Federal Regulations” (CFR) 40 CFR 261 of the United State. However, these three standards vary greatly in the classification and identification standards of hazardous waste [2]. The Basel Convention is similar to the Chinese list: Y3-Y45 are all related to industrial waste. European Waste Catalogue: chapter 03-17 and chapter 19 are all related to industrial waste. CFR is classified in different ways, F001-F039 and K001-K181 are all related to industrial waste. This paper takes the “National Hazardous Waste List” of China as the standard, because it studies hazardous waste disposal enterprises in China.

Because of the many different industries and the many different production techniques, there are many different types of hazardous waste with widely varying compositions and hazard levels, which means there are also great uncertainties in hazardous waste production, collection, transportation, storage, utilization and disposal. Therefore, controlling the pollution arising from hazardous industrial waste has become a major problem for enterprise environmental management and supervision. The “Law of the People’s Republic of China on the Environmental Protection of Solid Waste Pollution” states that hazardous waste must be disposed of only by enterprises that have been granted hazardous waste business licenses. Consequently, industrial waste enterprises need to outsource this work to third party hazardous waste disposal enterprises.

**Figure 1:**
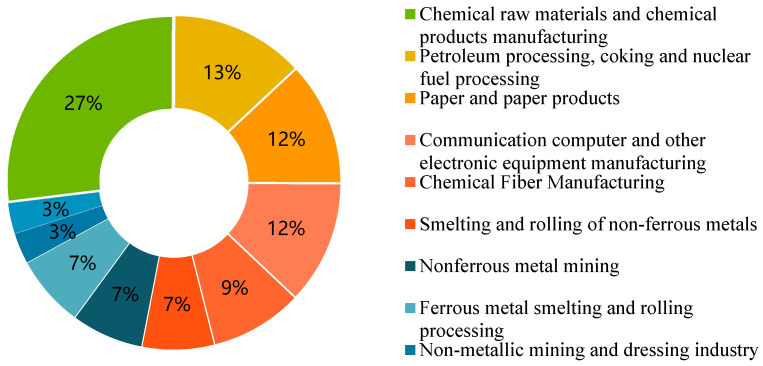
Hazardous waste in China.

As hazardous industrial waste is produced by many industries, it is complex to deal with (see Figure 1). Hazardous waste disposal provider selection is a multi-criteria decision making (MCDM) problem and an important part of enterprise management [3]. Generally, industrial enterprises have chosen third-party hazardous waste disposal contractors based on their professional experience and/or competitive bidding. However, as these decision-making processes often lack objective and scientific assessments, the most appropriate disposal enterprise may not be selected [4].

Therefore, the purpose of this paper is to develop an effective, efficient hazardous waste disposal enterprise evaluation system to assist industrial enterprises select the best contractor for their specific hazardous waste problems.

## 2. Literature Review 

The rapid growth in hazardous industrial waste is directly related to increased urbanization and technological development. Even though hazardous waste management has a significant impact on the economy and the environment, most previous waste management research has focused on general solid waste management, and as hazardous waste management research has only recently commenced in China, there have been relatively few papers published. 

Although hazardous waste disposal laws and regulations differ greatly from the treatment of general solid waste, lessons can still be drawn from solid waste disposal research. Eskandari divided the main criteria used to evaluate solid waste disposal programs into five different groups: finance, the environment, health and safety, community perception and life [5]. Arikan used PROMETHEE and fuzzy TOPSIS (Technique for Order Preference by Similarity to an Ideal Solution) to select the most feasible solid waste treatment technology for the existing scheme [6]. 

The hazardous waste industry is a multi-cross field industry that can be divided into hazardous medical waste, hazardous industrial waste and hazardous social waste. Hazardous medical waste has been widely studied. Karamouz proposed an assessment framework for the management of hospital solid waste [7]. As the selection of suitable hazardous waste transport enterprises involves many influencing factors, uncertainty, and inaccurate/incomplete data, we proposed a more effective selection model by combining the Delphi method, VIKOR (Vlse Kriterijumska Optimizacija I Kompromisno Resenje) and fuzzy set theory [8]. Although hazardous industrial waste is different from hazardous medical waste and hazardous social waste, similar aspects can still be used for reference.

Hsu developed an index system for infectious disease medical waste disposal companies that detailed many of the micro services but failed to consider the macro aspects, such as environmental protection, health, and sustainability [9]. Faisal established five first-class indicators and 23 s-class indicators for an evaluation index system for infectious disease medical waste disposal enterprises [10]; however, less focus was placed on special professional hazardous waste indicators, the index system seemed more applicable to the selection or evaluation of ordinary companies rather than being specific to hazardous waste disposal companies. Korkmazer determined that there were three standards: flexibility, risk and environmental risk, and 12 sub standards for hazardous waste disposal company selection; however, the indicator system was not comprehensive enough and influencing factors associated with price and procedure compliance were not considered [11]. Therefore, as no comprehensive index system has been developed based on recognized standards, applicable models are needed [12].

Most hazardous waste disposal enterprises (HWDE) selection studies have adopted an analytic hierarchy process (AHP) to compare two indexes and come to a conclusion; however, AHP has been found to have the following shortcomings.

(1) The value judgments are not reliable: AHP uses full compensation, that is, the high score for the target to be selected under some attributes makes up for the low scores for other attributes, which means that the final comprehensive weight does not fully reflect the actual situations for the projects/enterprises to be selected. However, as the PROMETHEE is not fully compensatory, the final evaluation results better reflect the real situations at the projects/enterprises to be selected [13].
(2) Large data input quantities are needed: AHP requires a significant quantity of system related data; however, PROMETHEE only needs a small data input quantity [13].
(3) The AHP pairs comparisons [14] suggest using the numbers 1 ~ 9 and their reciprocals as the scale; however, this had been found to be difficult to achieve in practice; however, there are no such artificial restrictions in the PROMETHEE.

Therefore, as a more practical and accurate approach, this paper used a hesitant fuzzy linguistic set so that the decision makers could provide their evaluations using several linguistic terms or comparative linguistic expressions, and then used the PROMETHEE to sort the HWDE [15,16].

Because of limited knowledge or background differences between decision makers, there are often decision-making judgment differences and uncertainties in group qualitative evaluations, which makes it impossible to determine a clear evaluation value for paired comparisons. Therefore, the classic PROMETHEE was extended to a fuzzy environment. Gupta combined fuzzy set theory with the PROMETHEE for greater flexibility [17], and Wang and Yang used a hybrid integration of PROMETHEE II and AHP to rank information systems and analyze the relationships between standards [18]. Tuzkaya integrated the Fuzzy Analytical Network Process (FANP) and F-PROMETHEE to select material handling equipment [19]. Chen proposed a preference ranking organization method (the fuzzy PROMETHEE) for a fuzzy affluence evaluation that assist decision makers or organizations improve their IS/IT outsourcing decision-making efficiency [20]. 

Therefore, compared with previous studies, this paper has the following innovations.

(1) This paper focuses on hazardous industrial waste, takes the hazardous waste producing enterprises as the reference objects, and designs a set of selection models for hazardous waste disposal enterprises to assist industrial waste producing enterprises rank alternative third-party enterprises.
(2) Based on previous research, a comprehensive, professional indicator system is developed for the selection of hazardous waste disposal enterprises for industrial waste enterprises, and a new scientific indicator system proposed.
(3) For the first time, this paper combined an AHP-PROMETHEE and hesitant fuzzy linguistics set to select hazardous industrial waste disposal enterprises, establish an effective industrial hazardous waste disposal enterprise evaluation index, and develop a new, more effective decision-making framework for disposal enterprise selection.

## 3. Evaluation Index System for Hazardous Waste Disposal Enterprises (EISHWDE)

### 3.1. Method Flow

This paper divided the selection of hazardous waste disposal enterprises into three levels: a target level to choose the suitable hazardous waste disposal enterprises; a scheme level to choose the three most suitable hazardous waste disposal enterprise alternatives in Sichuan Province, China, which are nominated *a*_1_, *a*_2_ and *a*_3_ for privacy reasons; and index levels based on six first-level indicator—service ability, reliability, cost, price, additional services, environment and safety—and several second-level indicators. 

**Figure 2:**
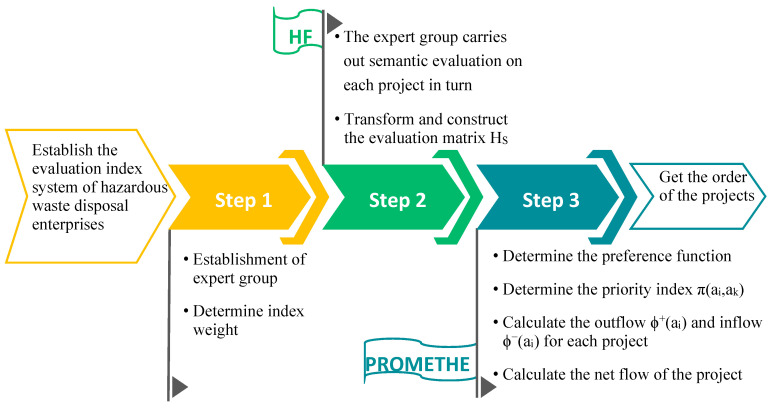
Logical structure for the hesitant fuzzy linguistic (HFL)-PROMETHEE model.

The calculation process was divided into three main stages. In the first stage, the scientific evaluation index system was established based on the hazardous waste scene, and an expert group formed to calculate the weights for each index. In the second stage, the expert group scored the three enterprises for each attribute, after which a hesitant fuzzy linguistic (HFL) evaluation matrix was constructed. In the third stage, the preference functions were determined, the net flow calculated, and after the scheme sorting, the final results were obtained. Figure 2 gives the logical structure diagram for the model. 

### 3.2. Index System Construction

When choosing a hazardous waste disposal enterprise, the first thing is that the enterprise should have the qualification to dispose hazardous waste, otherwise it is illegal in China. Research on the selection of hazardous-waste-related enterprises has been mainly focused on transportation enterprise selection and medical waste disposal enterprises; however, no reasonable evaluation and selection index system for hazardous industrial waste disposal enterprises has yet been developed. Therefore, based on previous research and field investigations, this paper established a more scientific EISHWDE and added a secondary index. As shown in Table 1.

This paper starts from the concept of hazardous waste, takes supplier selection as the target, and considers all factors. According to Swift [27], five factors are important when selecting waste disposal suppliers: Product, Reliability, Experience, Price and Availability. From this, three first-level indicators were obtained in this paper: capability, reliability and price. At the same time, based on the studies of other scholars on the choice of disposal providers for general solid waste, medical waste and hazardous waste. In combination with practical learning, the article extracted three other first-level indicators: cost, additional capability and environment.

**Service capability:** The most important factor for industrial waste enterprises (IWEs) is the service capabilities of the HWDE, that is, what the supplier can provide to the enterprise, as the main problem that the IWE wants to solve is to remove the hazardous waste from the plant and dispose of it legally. At the same time, from the perspective of the HWDE, enterprise waste storage and disposal capacities are particularly important; therefore, the higher these two capacities, the more secure the services the IWE can receive. Related to this are the HWDE equipment and technical capabilities in terms of size, sophistication and effectiveness as technical capability is a guarantee of hazardous waste disposal effectiveness and speed [26]. HWDE service quality in terms of communication, waste disposal, goods handling, recording, and service flexibility also affect the overall service experience of IWE and determine whether the cooperative process is smooth.

**Reliability:** As HWDE selection is a single enterprise decision, product reliability is an important factor for IWE management Swift as the management, organization and financial status of the HWDE ensures smooth operations for the IWE, which includes rigorous procedural compliance to ensure the legality of the IWE hazardous waste disposal; therefore, industry reputation and performance history are key reference when evaluating HWDEs [28].

**Cost:** Unit disposal costs refer to the disposal costs incurred by the disposal company when disposing of one tonne of waste, and unit transportation cost refers to the cost of transferring the hazardous waste from the IWE to the disposal company [11]. As cost is usually proportional to price, it is necessary to consider cost when considering price.

**Price:** In addition to reliability, price is an important criterion for selecting a third-party contractor, as the IWE needs to ensure that the price is within an estimated range.

**Additional services:** These days, effective and efficient information systems are vital for efficient management. Management information systems provide information about enterprise vehicles, personnel, equipment, and operations, and also improve IWE management efficiency as it provides a clear, real-time source of information about the HWDE disposal process. 

In this paper, a new index that involves inter-provincial trade is proposed because: ① as part of their niche production, IWEs often produce small quantities of special types of hazardous waste that may not be able to be processed in the local province, which means the waste may need to be transferred across the province. ② If the HWDE treatment capacity is insufficient, the hazardous waste warehouse is full, or they are unable to receive the waste on time, the HWDE could join with another HWDE to find a solution. However, these types of cross-provincial transactions are not a requirement and are therefore an additional possible HWDE service. As the recipient of the hazardous waste, each HWDE guarantees hazardous waste transportation and storage safety to prevent accidents or leakage; therefore, corresponding emergency capacity is also essential.

**Environment and safety:** The training of disposal workers in the basics of hazardous waste disposal includes aspects related to public health and environmental pollution, that is, the health and environmental impacts of hazardous waste and preventive measures [26]. Before selecting a contractor, therefore, the potential environmental liability issues need to be thoroughly reviewed by assessing and considering the HWDE’s sustainability plan [25].

### 3.3. Index Weight Determinations

As the PROMETHEE does not give a specific method to determine the indicator weights and requires decision makers to generate their own weights based on the actual situation, the weight determination is more subjective [13]. Therefore, AHP is needed to compare the evaluation criteria, and the geometric average method applied to determine the weights.

**Figure 3:**
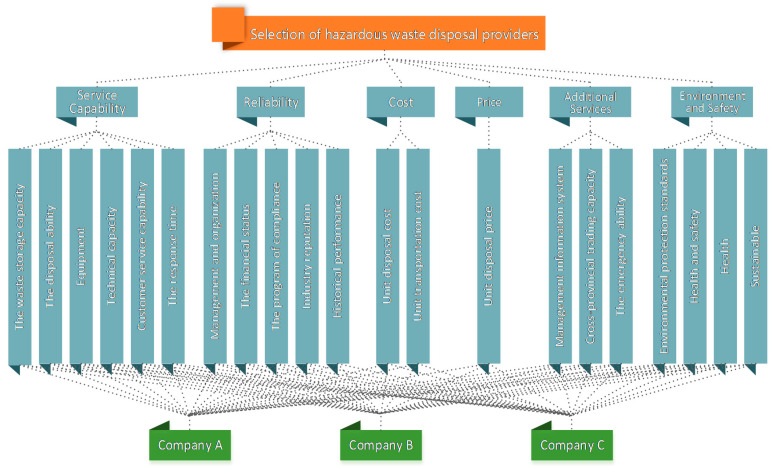
Hierarchical structure for hazardous waste disposal enterprise selection.

Step 1: Based on the six first-level indicators and 21 s-level indicators proposed in this paper, the hierarchical structure for the hazardous waste disposal enterprise selection was obtained, as shown in Figure 3.

Step 2: As the proportions for each indicator in the target measurement are not necessarily the same, the decision maker first selects the indicator elements based on the hazardous waste disposal enterprise evaluation, compares them, and obtains the quantitative results, from which the judgment matrix *A* = (*a_ij_*)*_m × m_*, *m* = 1,2,…,21 is developed.

Step 3: A through to *Aω* = *λ_max_*(*A*), *ω* being the normalized weights *ω*, *ω* = (*ω*_1_, *ω*_2_, …, *ω*_m_)^T^, are exported, where *λ_max_*(*A*) is the largest eigenvalue for *A*, the results for which are shown in Table 2.

Step 4: Calculate the consistency ratio *R_c_*(*A*):

(1) $$R_{c}(A)=\frac{\lambda_{\max}\left(A\right)-m}{(m-1)I_{R}}$$

where *I_R_* is a random consistency index. As the calculated CR < 0.10, the judgment consistency matrix is deemed acceptable. Therefore, the weight of the m evaluation indexes is *ω* = (0.0812, 0.0774, 0.0774, 0.1389, 0.0205, 0.0247, 0.0225, 0.0297, 0.0560, 0.0869, 0.0424, 0.0164, 0.0328, 0.1559, 0.0101, 0.0027, 0.0148, 0.0116, 0.0202, 0.0285, 0.0494)^T^, where T is the transpose of the matrix.

## 4. Methodology

### 4.1. Basic Concepts

**Definition** **1.** *Set S = {s_ɡ_|ɡ = 0,1,…,τ} as the linguistic term set, with the hesitant fuzzy linguistic term set being H_S_:*

(2) $$H_{S}=\left\{<a_{i},h_{S}(a_{i})>|a_{i}\in A\right\}$$

*where a_i_ ∈A, I = 1,2,…,N. A→S is the possible membership degree for element a_i_∈A mapped to set X⊂A, and h_S_(a_i_) is a continuous possible value in the linguistic term S, and satisfies h_S_(a_i_) = {s_φl_(a_i_)|s_φl_(a_i_)∈S, l = 1,…, L(a_i_)}, where φl ∈ {0,1,…,τ}, L(a_i_) is the number of linguistic terms in h_S_(a_i_). In summary, h_S_(a_i_) is the number of hesitant fuzzy linguistic terms, and H_S_ is the set of all hesitant fuzzy linguistic terms in linguistic term S. *

**Definition** **2.** *Set S as the linguistic term set and G_H_ as the text-free grammar. Define G_H_ = (V_N_,V_T_,I,P), where V_N_ = {subject, compound word, unitary relation, binary relation, conjunction}; V_T_ ={“less than”, “more than”, “at least”, “at most”, “in… Between ”, “and”, “s_0_”, “s_1_”, …, “s_τ_”}; I ∈V_N_; P = {I refers to subject or compound word; subject refers to “ s_0_”, “ s_1_”,…, “ s_τ_”; compound words refer to unitary relation + subject or dualistic relation + conjunction + subject; unitary relation means “less” or “more”, and dualistic relation means “in…between”; and the conjunction is “and”}.*

**Definition** **3.** *Suppose $E_{G_{H}}$ can transform the G_H_ generated linguistic expression ll∈S_ll_ into H_S_, then S is the linguistic term set adopted by G_H_ and S_ll_ is the set of all expressions generated by G_H_. The linguistic expression generated by G_H_ can be converted into the hesitant fuzzy linguistic set using the following formula:*

$E_{G_{H}}$: *S_ll_*→*H_S_*;

- $E_{G_{H}}$*(s_α_) = {s_ɡ_∣s_ɡ_ ∈ S};*
- $E_{G_{H}}$*(at most s_α_) = {s_ɡ_∣s_ɡ_ ∈ S and s_ɡ_ ≤ s_α_};*
- $E_{G_{H}}$*(less than s_α_) = {s_ɡ_∣s_ɡ_ ∈ S and s_ɡ_ < s_α_};*
- $E_{G_{H}}$*(at least s_α_) = {s_ɡ_∣s_ɡ_ ∈ S and s_ɡ_ ≥ s_α_};*
- $E_{G_{H}}$*(more than s_α_) = {s_ɡ_∣s_ɡ_ ∈ S and s_ɡ_ > s_α_};*
- $E_{G_{H}}$*(between s_α_ and s_β_) = {s_ɡ_∣s_ɡ_ ∈ S and s_α_ ≤ s_ɡ_ ≤ s_β_};*

**Definition** **4.** *For each HFLTS, according to Definition 1, we can obtain the lower bound*$h_{S}^{\mathrm{ij}-}=\min_{l=1,\ldots ,\#h_{S}^{ij}}\left\{s_{\delta_{l}^{ij}}\right\}$*and the upper bound*$h_{S}^{\mathrm{ij}+}=m{\mathrm{ax}}_{l=1,\ldots ,\#h_{S}^{ij}}\left\{s_{\delta_{l}^{ij}}\right\}$. *Then, we can define the notions of the hesitant fuzzy linguistic positive ideal solution A^+^ and hesitant fuzzy linguistic negative ideal solution A^−^ as follows, respectively: A^+^ = {*$h_{s}^{1+}$*,*$h_{s}^{2+}$*, …,*$h_{s}^{n+}$*} and A^−^ = {*$h_{s}^{1-}$*,*$h_{s}^{2-}$*, …,*$h_{s}^{n-}$*}, of which,*

(3) $$\begin{array}{c} h_{s}^{j+}=\left\{ \begin{array}{l} \underset{i=1,2,3,\ldots ,m}{\max}h_{s}^{ij+}=\underset{\begin{array}{l} i=1,2,3,\ldots ,m\\ l=1,\ldots ,\#h_{s}^{ij} \end{array}}{\max}\left\{s_{\delta_{l}^{{}^{ij}}}\right\},{\mathrm{for}\;\mathrm{benefit}\;\mathrm{criterion}\;c}_{j}\;\\ \underset{i=1,2,3,\ldots ,m}{\min}h_{s}^{ij-}=\underset{\begin{array}{l} i=1,2,3,\ldots ,m\\ l=1,\ldots ,\#h_{s}^{ij} \end{array}}{\min}\left\{s_{\delta_{l}^{{}^{ij}}}\right\},{\mathrm{for}\;\cos t\;\mathrm{criterion}\;c}_{j} \end{array}\right.\\ h_{s}^{j-}=\left\{ \begin{array}{l} \underset{i=1,2,3,\ldots ,m}{\max}h_{s}^{ij+}=\underset{\begin{array}{l} i=1,2,3,\ldots ,m\\ l=1,\ldots ,\#h_{s}^{ij} \end{array}}{\max}\left\{s_{\delta_{l}^{{}^{ij}}}\right\},{\mathrm{for}\;\cos t\;\mathrm{criterion}\;c}_{j}\\ \underset{i=1,2,3,\ldots ,m}{\min}h_{s}^{ij-}=\underset{\begin{array}{l} i=1,2,3,\ldots ,m\\ l=1,\ldots ,\#h_{s}^{ij} \end{array}}{\min}\left\{s_{\delta_{l}^{{}^{ij}}}\right\},{\mathrm{for}\;\mathrm{benefit}\;\mathrm{criterion}\;c}_{j} \end{array}\right. \end{array}$$

*for j = 1,2,…,m.*

*In order to choose the desired alternative, we can calculate the distance between each alternative A_j_ and the hesitant fuzzy linguistic positive ideal solution A^+^, and the distance between each alternative A_j_ and the hesitant fuzzy linguistic negative ideal solution A^−^, respectively. Intuitively, the smaller the distance d(A_j_,A^+^), the better the alternative; while the larger the distance d(A_j_,A^−^), the better the alternative.*

### 4.2. Hesitant Fuzzy Linguistic PROMETHEE (HFL-PROMETHEE)

There are two forms of evaluation information in decision matrices when dealing with multi-criteria decision making: qualitative information and quantitative information. Generally, as most real production problems lack quantitative information, qualitative information is particularly important. Because of the complexity of the HWDE selection and the different expert backgrounds and experience, there is significant expert uncertainty and ambiguity in the evaluation process; however, this can be reduced using HFL term sets. When these are combined with the evaluation sequence construction abilities in the PROMETHEE, a scientific and effective model for IWE decision makers for HWDE selection can be built. The specific steps in the PROMETHEE are described in the following.

1. Build the evaluation function matrix.

Step 1. Define the multi-criteria decision problem: let A = {*a*_1_,*a*_2_,…,*a_n_*} be the scheme set composed of n schemes, C = {*c*_1_,*c*_2_,…,*c_m_*} for *m* indexes for the index set, and ω = {*ω*_1_,*ω*_2_,…,*ω_m_*}^T^ for each index weight so that 0 ≤ *ω_j_* ≤ 1 and $\sum_{j=1}^{m}\omega_{j}=1$.

Step 2. An expert group is formed for this multi criteria decision-making problem, with the qualitative evaluation of each index in each scheme using linguistic term set *S* and text-free grammar *G_H_*, so that a final linguistic expression, ll, is generated.

Step 3. $E_{G_{H}}$ transforms the linguistic expression *ll* into *H_S_*, and new linguistic terms are added until the number of linguistic terms in each *H_S_* is the same to make it more convenient for calculation.

2. Determine the preference function.

Step 4. The fuzzy linguistic number $h_{s}^{ij}=\{s_{\delta l}^{ij}|l=1,\ldots ,\#h_{s}^{ij}\}(i=1,2,\ldots ,m;j=1,2,n)$ represents the satisfaction degree of the scheme *a_i_* on index **c*_j_*. Make the sum of all the hesitant fuzzy linguistic terms in each hesitant fuzzy linguistic set $\sigma_{s}^{ij}=\sum_{l=1}^{\#h_{s}^{ij}}\delta_{l}^{ij}$. For index **c*_j_*, the deviation between *a_i_* and *a_k_* for any pair of schemes is:

(4) $$d_{j}(a_{i},a_{k})=\sigma_{s}^{ij}-\sigma_{s}^{kj},(i,k=1,2,\ldots ,n)$$

Step 5. Determine the positive ideal solution $A_{j}^{+}$ and the negative ideal solution $A_{j}^{-}$ under index *c*_j_, and calculate the deviation *d_j_*($A_{j}^{+}$,$A_{j}^{-}$) for the positive and negative ideal solutions.

Step 6. For the linear preference criterion function, take the preference threshold v = *θ* *d_j_*($A_{j}^{+}$,$A_{j}^{-}$), 0 < *θ* < 1, with the decision maker selecting a value for *θ* based on need. When |*f(a_i_) − f(a_k_) | = 0*, scheme *a_i_* and scheme *a_k_* are no different, and when $\left|f(a_{i})-f(a_{k})\right|>\theta d_{j}(A_{j}^{+},A_{j}^{-})$, scheme *a_i_* is stricter than scheme *a_k_*. Therefore, the linear criterion preference function is:

(5) $$\begin{array}{l} P_{j}(a_{i},a_{k})=\{\begin{array}{cc} 0, & d_{j}(a_{i},a_{k})\leq 0\\ \frac{d_{j}(a_{i},a_{k})}{\theta d_{j}(A_{j}^{+},A_{j}^{-})}, & 0<d_{j}(a_{i},a_{k})\leq \theta d_{j}(A_{j}^{+},A_{j}^{-})\\ 1, & d_{j}(a_{i},a_{k})\geq \theta d_{j}(A_{j}^{+},A_{j}^{-}) \end{array}\\ 0<\theta <1 \end{array}$$

3. Priority function.

Step 7. Priority index *π*(*a_i_, a_k_*) indicates the degree to which scheme *a_i_* is superior to scheme *a_k_*, with the closer the result to 1, the higher the superiority of scheme *a_i_*.

(6) $$\pi (a_{i},a_{k})=\sum_{r=1}^{m}\omega_{j}P_{j}(a_{i},a_{k})$$

*J* = 1,2,…,*m*; *i*,*k* = 1,2,…,*n*.

4. Calculate the outflow and inflow for each scheme.

Step 8. Based on the priority index, calculate the outflow *φ*^+^(*a_i_*) and inflow *φ*^−^(*a_i_*) for each scheme.

Outflow:

(7) $$\varphi^{+}(a_{i})=\sum_{r=1}^{m}\pi (a_{i},a_{k})=\sum_{j=1}^{n}\sum_{r=1}^{m}\omega_{j}P_{j}(a_{i},a_{k})$$

Inflow:

(8) $$\varphi^{-}(a_{i})=\sum_{r=1}^{m}\pi (a_{k},a_{i})=\sum_{j=1}^{n}\sum_{r=1}^{m}\omega_{j}P_{j}(a_{k},a_{i})$$

*J* = 1,2,…,*m*; *i*, *k* = 1,2,…,*n*.

Where *φ*^+^(*a_i_*) represents the preference priority degree for scheme *a_i_;* the greater the value, the higher the degree of superiority of scheme *a_i_* over all other schemes; and *φ*^−^(*a_i_*) indicates the degree to which other schemes have preference over other schemes; the smaller the value, the higher the degree to which scheme *a_i_* is superior to other schemes.

5. Calculate the net flow of the scheme.

Step 9. Calculate the net flow of scheme *a_i_*.

Net flow:

(9) $$\varphi (a_{i})=\varphi^{+}(a_{i})-\varphi^{-}(a_{i})$$

The greater the value of *φ*(*a_i_*), the higher the goodness of scheme *a_i_*; therefore, *φ*(*a_i_*) > *φ*(*a_k_*)indicates that scheme *a_i_* is superior to scheme *a_k_*. By analogy, the full order of the scheme to be selected can then be obtained.

## 5. Results and Discussion

This paper is based on the industrial resources of the National Hazardous Waste Disposal Engineering Technology Center. Professionals in the industry were interviewed and invited to form a panel of experts. The case study was based on a chemical enterprise in Chengdu, Sichuan province, China, and was evaluated and ranked by three HWDEs. The details are not given because the information involves the privacy of the enterprise.

### 5.1. First Stage

**Step 1: Establish the HWDE evaluation index system.**

Combined with the hazardous waste industry characteristics, the HWDE evaluation index system was established as explained in the Section 3 of this paper and as shown in Table 1. This system improved the research for HWDE selection and enriched and updated modern HWDE evaluation standards.

**Step 2: Create an expert group.**

Besides the establishment of *a*_1_,*a*_2_,*a*_3_, three IWE waste management directors or one director and two industry experts were selected to form an expert group based on the following criteria: ① at least 5 years of experience in the management of hazardous waste disposal; ② experience in evaluating and selecting HWDE [22].

**Step 3: Determine the index weight.**

In Section 3, AHP was used to calculate the weights for all secondary indexes and passed the consistency test, with the final weights for the m indexes being ω = (0.0812, 0.0774, 0.0774, 0.1389, 0.0205, 0.0247, 0.0225, 0.0297, 0.0560, 0.0869, 0.0424, 0.0164, 0.0328, 0.1559, 0.0101, 0.0027, 0.0148, 0.0116, 0.0202, 0.0285, 0.0494)^T^.

### 5.2. Second Stage

**Step 1: Build linguistic term set S.**

The linguistic term set *S* for the 21 indicators given in this paper was expressed as *S* = {*s*_0_ = terrible, *s*_1_ = worse, *s*_2_ = bad, *s*_3_ = medium, *s*_4_ = good, *s*_5_ = better, *s*_6_ = perfect}.

**Step 2: Expert group conducts a semantic evaluation of each solution in turn.**

The expert group qualitatively evaluated *a*_1_, *a*_2_, and *a*_3_ enterprises against each indicator by referring to linguistic term set S. As all evaluations were based on subjective expert judgments, the opinions were inconsistent at times; for example, if all members of the expert group agreed that enterprise *a*_1_ performed better in terms of emergency capability, the assessment information was expressed as {*s*_4_}; however, if one expert thought that enterprise *a*_1_ performed moderately for emergency capability, and another expert thought that enterprise *a*_1_ performed at least moderately or above for emergency capability and they could note persuade each other to change their preferences, then the evaluation information was expressed as {*s*_3_, *s*_4_, *s*_5_, *s*_6_}. After discussion and scoring by the expert group, the semantic evaluations for all 21 indicators for all three HWDE were conducted, and the qualitative evaluation information table obtained (due to the space restrictions, it is not shown here).

**Step 3: Transform and construct the hesitant fuzzy linguistic evaluation matrix *H_S_*.**

Using the transformation function $E_{G_{H}}$ in Definition 3, the linguistic expression ll in the qualitative evaluation information table was transformed and the hesitant fuzzy linguistic evaluation matrix *H_S_* constructed:

$$\begin{array}{l} \begin{array}{c} a_{1}\\ a_{2}\\ a_{3} \end{array}\begin{array}{c} {}[ \end{array}\begin{array}{ccccccccccc} c_{1} & c_{2} & c_{3} & c_{4} & c_{5} & c_{6} & c_{7} & c_{8} & c_{9} & c_{10} & c_{11}\\ \left\{s_{4},s_{5}\right\} & \left\{s_{2},s_{3},s_{4}\right\} & \left\{s_{2},s_{3}\right\} & \left\{s_{2},s_{3}\right\} & \left\{s_{2}\right\} & \left\{s_{2},s_{3}\right\} & \left\{s_{3}\right\} & \left\{s_{3},s_{4}\right\} & \left\{s_{4},s_{5}\right\} & \left\{s_{3}\right\} & \left\{s_{3}\right\}\\ \left\{s_{2}\right\} & \left\{s_{2},s_{3},s_{4}\right\} & \left\{s_{3}\right\} & \left\{s_{3},s_{4}\right\} & \left\{s_{2},s_{3},s_{4}\right\} & \left\{s_{2},s_{3}\right\} & \left\{s_{3},s_{4}\right\} & \left\{s_{4}\right\} & \left\{s_{4},s_{5}\right\} & \left\{s_{3},s_{4}\right\} & \left\{s_{4},s_{5}\right\}\\ \left\{s_{3},s_{4}\right\} & \left\{s_{3},s_{4}\right\} & \left\{s_{4},s_{5}\right\} & \left\{s_{4},s_{5}\right\} & \left\{s_{2},s_{3},s_{4}\right\} & \left\{s_{2}\right\} & \left\{s_{2},s_{3},s_{4}\right\} & \left\{s_{3},s_{4}\right\} & \left\{s_{3}\right\} & \left\{s_{3}\right\} & \left\{s_{3},s_{4}\right\} \end{array}\begin{array}{c} ] \end{array}\\ \begin{array}{c} a_{1}\\ a_{2}\\ a_{3} \end{array}\begin{array}{c} {}[ \end{array}\begin{array}{cccccccccc} c_{12} & c_{13} & c_{14} & c_{15} & c_{16} & c_{17} & c_{18} & c_{19} & c_{20} & c_{21}\\ \left\{s_{2},s_{3}\right\} & \left\{s_{3}\right\} & \left\{s_{2},s_{3}\right\} & \left\{s_{2},s_{3}\right\} & \left\{s_{1},s_{2}\right\} & \left\{s_{3},s_{4}\right\} & \left\{s_{3},s_{4}\right\} & \left\{s_{3},s_{4}\right\} & \left\{s_{3}\right\} & \left\{s_{2},s_{3}\right\}\\ \left\{s_{3},s_{4}\right\} & \left\{s_{3}\right\} & \left\{s_{4}\right\} & \left\{s_{3},s_{4}\right\} & \left\{s_{3},s_{4}\right\} & \left\{s_{2},s_{3},s_{4}\right\} & \left\{s_{3}\right\} & \left\{s_{2},s_{3},s_{4}\right\} & \left\{s_{3}\right\} & \left\{s_{3},s_{4}\right\}\\ \left\{s_{4},s_{5}\right\} & \left\{s_{3},s_{4}\right\} & \left\{s_{3},s_{4}\right\} & \left\{s_{3}\right\} & \left\{s_{1},s_{2}\right\} & \left\{s_{3},s_{4},s_{5}\right\} & \left\{s_{3},s_{4}\right\} & \left\{s_{3}\right\} & \left\{s_{2},s_{3}\right\} & \left\{s_{2},s_{3},s_{4}\right\} \end{array}\begin{array}{c} ] \end{array} \end{array}$$

In subsequent calculations, the linguistic terms needed to be added to the current evaluation matrix so that the number of linguistic terms in each hesitant fuzzy linguistic set in the matrix remained the same. For example, {*s*_3_} was supplemented as {*s*_3_, *s*_3_, *s*_3_}, and {*s*_3_, *s*_4_} as {*s*_3_, *s*_7/2_, *s*_4_} (due to space restrictions, this is not shown here).

### 5.3. Third Stage

**Step 4: Determine the positive ideal solution A^+^ and the negative ideal solution A^−^ and obtain the deviation** $d_{j}(A_{j}^{+},A_{j}^{-})$ **.**

In the index system, the IWE response time (*c*_6_), unit transportation costs (*c*_12_), unit transportation price (*c*_13_) and product costs (*c*_14_) make up the cost indexes, with the remainder being the benefit indexes. The positive ideal solution A^+^ and the negative ideal solution A^−^ for the hesitant linguistics terms and the deviation $d_{j}(A_{j}^{+},A_{j}^{-})$ between them were then calculated separately, with the results being as follows:

Positive ideal solution: A^+^ = {*s*_5_,*s*_4_,*s*_5_,*s*_5_,*s*_4_,*s*_1/2_,*s*_4_,*s*_4_,*s*_5_,*s*_4_,*s*_5_,*s*_1/2_,*s*_1/3_,*s*_1/2_,*s*_4_,*s*_4_,*s*_5_,*s*_4_,*s*_4_,*s*_3_,*s*_4_};

Negative ideal solution: A^−^ = {*s*_2_,*s*_2_,*s*_2_,*s*_2_,*s*_2_,*s*_1/3_,*s*_2_,*s*_3_,*s*_3_,*s*_3_,*s*_3_,*s*_1/5_,*s*_1/4_,*s*_1/4_,*s*_2_,*s*_1_,*s*_2_,*s*_3_,*s*_2_,*s*_2_,*s*_2_};

Deviation: $d_{j}(A_{j}^{+},A_{j}^{-})=\left\{\frac{27}{5},\frac{18}{5},\frac{27}{5},\frac{27}{5},\frac{18}{5},\frac{2}{7},\frac{18}{5},\frac{9}{5},\frac{18}{5},\frac{9}{5},\frac{18}{5},\frac{5}{9},\frac{1}{7},\frac{4}{9},\frac{18}{5},\frac{27}{5},\frac{27}{5},\frac{9}{5},\frac{18}{5},\frac{9}{5},\frac{18}{5}\right\}$.

**Step 5: Determine the preference function.**

The degree to which scheme *a_i_* was superior to scheme *a_k_* was calculated using the linear criterion preference function in Formula (5), the results for which are shown in Table 3 (θ = 0.6 based on the decision facts and the IWE’s preference for strict superiority).

**Step 6: Calculate the priority index *π*(*a_i_*,*a_k_*)**

Using Formula (6), the calculated priority function was determined, as shown in Table 4.

**Step 7: Calculate the outflow *φ*^+^(*a_i_*) and inflow *φ*^−^(*a_i_*) for each scheme.**

Using Formulas (7) and (8), *φ*^+^(*a_i_*) and *φ*^−^(*a_i_*) were calculated for each scheme, as shown in Table 5.

**Step 8: Calculate the flow *φ*(*a_i_*) for each scheme.**

Using Formula (9), the net flow *φ*(*a_i_*) was calculated for each scheme. The result was: *φ*(*a*_1_) = −0.7452, *φ*(*a*_2_) = 0.5066, *φ*(*a*_3_) = 0.2386; therefore, after the evaluation and calculation process, the full ranking for the three IWEs was determined to be *a*_1_ < *a*_3_ < *a*_2_; that is, the comprehensive performance of enterprise *a*_2_ was the best, followed by enterprise *a*_3_, with enterprise *a*_1_ being the least ideal.

### 5.4. Sensitivity Analysis

**Figure 4:**
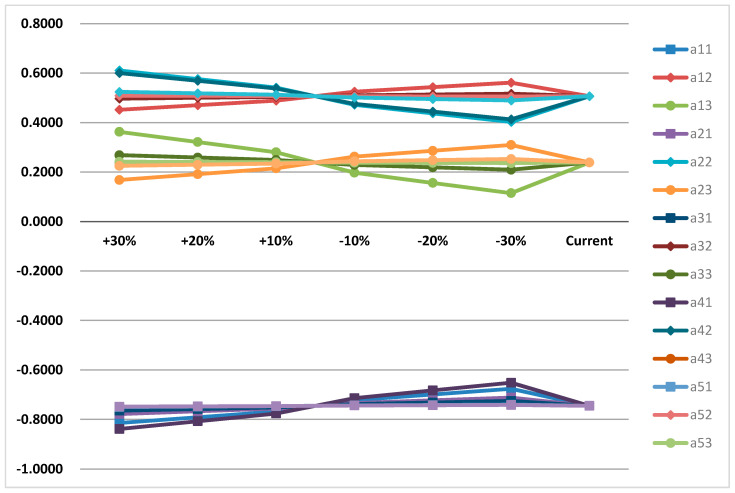
Sensitivity analysis was carried out according to the weight change of each level index.

A sensitivity analysis is employed to verify the reliability and feasibility of models or methods. To examine the robustness of the preference ranking among the HWDE. In this study, a factor’s weight was increased and reduced equally to assess the reliability of the system. Figure 4 sensitivity analysis according to the weight changes of service capability (*a*_1*i*_), reliability (*a*_2*i*_), cost (*a*_3*i*_), price (*a*_4*i*_), additional services (*a*_5*i*_), environment and safety (*a*_6*i*_), I = 1,2,3 (“i” stands for enterprise *a*_1_, *a*_2_, *a*_3_). The scenario for each weight is presented in Figure 4 by adjusting the weight of each criteria to +30%, +20%, +10%, −10%, −20%, −30%, respectively.

By comparing the alternatives according to the changes of six different weights as shown in the figure, there is no difference in ranks for the HWDE selection. So, the sensitivity analysis shows that the results are very sensitive to the possible errors and provide reliable results.

### 5.5. Discussion

The outsourcing of hazardous waste disposal has become more common in China because most enterprises do not have the necessary hazardous waste licenses and outsourcing also allows the enterprises to focus on their main businesses. In theory, industrial waste enterprises can reduce or minimize their risks by collecting their hazardous waste based on government regulations and outsourcing the disposal work to qualified suppliers. However, outsourcing is not entirely risk-free.

In previous studies on hazardous waste disposal enterprise selection, evaluation indexes have only been developed for individual medical infectious waste, with the evaluation index systems often being little more that adaptations of general supplier selection criteria, which meant that the special hazardous waste disposal requirements in special industries were not considered. Therefore, based on the six dimensions of service capability, reliability, cost, price, additional services and environment and safety, this paper refined the hazardous waste disposal evaluation content to comprehensively include special hazardous waste requirements. Furthermore, because some niche industries produce small quantities of a specific types of hazardous waste, there may be no suitable disposal enterprise in their province; therefore, this paper suggested an “inter-provincial transaction capacity” index be added to symbolize the service scope of the disposal enterprise and reflect its waste storage capacity.

Hazardous waste disposal companies are often selected through oral negotiations or bidding decisions by different experts and managers. To ensure more suitable results, this paper constructed an HFL-PROMETHEE and AHP process that was able to resolve the semantic and expert scoring ambiguities using hesitant fuzzy linguistic terms to convert the qualitative information into quantitative information. This step converts the language information into a machine-operated format (digital information) in which calculations can be performed. According to different perspectives, different importance degrees after quantization are added, respectively, and the evaluation results of decision makers can be truly and comprehensively expressed in the form of figures.

The advantage of the proposed simple and scientific evaluation model for industrial production waste enterprises was that evaluation sequences could be constructed using small amounts of data to obtain the ranking results for the candidate options. The results of this study can be provided to any HW operator/manager without an engineering/mathematics degree. Using computer technology, all the algorithms and formulas of the model are built into a computer program. The decision maker evaluates the situation as it really is and the computer program gives the final result. The combination of AHP and PROMETHEE overcomes the limitations of a single method in selecting vendors, effectively increasing the objectivity and accuracy of evaluation. The sensitivity analysis in this paper shows that the system improves the accuracy and rationality of supplier selection results to a certain extent and has certain reliability.

## 6. Conclusions

Choosing appropriate hazardous waste disposal enterprises has often been subjective, especially for industrial production waste enterprise managers who lack standard and objective decision-making procedures and decision-making indicators. Therefore, this paper established an evaluation and selection index system suitable for Chinese hazardous waste disposal enterprises that included special hazardous waste industry standards in the general supplier selection criteria to ensure a professional, comprehensive index evaluation system. AHP was used to determine the index weights, with the results indicating that the selection of the most suitable hazardous waste disposal company was based on service capacity (0.4202), reliability (0.2374), cost (0.0492), price (0.1559), additional services (0.0276), and environment and safety (0.1097). The “Law of the People’s Republic of China on the Prevention and Control of Environmental Pollution by Solid Wastes” states that hazardous-waste-generating units must dispose of hazardous waste in accordance with relevant Chinese regulations and must entrust hazardous waste disposal to qualified enterprises. Therefore, to assess the service capabilities and reliability of hazardous waste disposal companies, this paper used an HFL-PROMETHEE method to establish the evaluation model. Hesitant fuzzy linguistics were applied to solve the uncertainties associated with the decision-making process, and the enterprise ranking was finally determined using the PROMETHEE. This study provides an efficient objective method for industrial waste companies to choose the most suitable hazardous waste disposal companies, and also provides solutions for decision makers who lack scientific and reasonable reference standards and cannot express their preferences accurately using numerical values.

## Figures and Tables

**Table 1:** Hazardous waste disposal enterprise selection evaluation index system.

	First-Level Index	Second-Level Index	Source
Evaluation and selection indicators of hazardous waste disposal enterprises	Service Capability (SC)	*c*_1_. Waste storage capacity	[21]
	Service Capability (SC) Reliability (R)	*c*_2_. Disposal capacity	[21]
		*c*_3_. Equipment	[9,22]
		*c*_4_. Technical capability	[22]
		*c*_5_. Customer service capability	[23]
		*c*_6_. Response time	[24]
		*c*_7_. Management and organization	[22]
	Reliability (R) Cost (Cost)	*c*_8_. Financial status	[22]
		*c*_9_. Procedural compliance	[22]
		*c*_10_. Industry reputation	[22]
		*c*_11_. Historical performance	[22]
		*c*_12_. Unit disposal cost	[11]
	Cost (Cost) Price (Price)	*c*_13_. Unit transportation cost	[11]
		*c*_14_. Unit disposal price	[9]
	Additional Services (AS)	*c*_15_. Management information systems	[10]
	Additional Services (AS) Environment and Safety (ES)	*c*_16_. Cross-provincial trading capacity	New
		*c*_17_. The emergency ability	[25]
		*c*_18_. Environmental protection standards	[26]
	Environment and Safety (ES)	*c*_19_. Health and safety	[24]
		*c*_20_. Health	[26]
		*c*_21_. Sustainability	[25]

**Table 2:** Index weights.

	SC						R				
ω	0.4202						0.2374				
	*c*_1_	*c*_2_	*c*_3_	*c*_4_	*c*_5_	*c*_6_	*c*_7_	*c*_8_	*c*_9_	*c*_10_	*c*_11_
ω	0.0812	0.0774	0.0774	0.1389	0.0205	0.0247	0.0225	0.0297	0.0560	0.0869	0.0424
	**Cost**		**Price**	**AS**			**ES**				
	0.0492		0.1559	0.0276			0.1097				
	*c*_12_	*c*_13_	*c*_14_	*c*_15_	*c*_16_	*c*_17_	*c*_18_	*c*_19_	*c*_20_	*c*_21_	
	0.0164	0.0328	0.1559	0.0101	0.0027	0.0148	0.0116	0.0202	0.0285	0.0494	

**Table 3:** Superiority degree for *a_i_* over *a_k_*.

	*c*_1_	*c*_2_	*c*_3_	*c*_4_	*c*_5_	*c*_6_	*c*_7_	*c*_8_	*c*_9_	*c*_10_	*c*_11_	*c*_12_	*c*_13_	*c*_14_	*c*_15_	*c*_16_	*c*_17_	*c*_18_	*c*_19_	*c*_20_	*c*_21_
*P_j_*(*a*_1_,*a*_2_)	1	0	0	0	0	0	0	0	0	0	0	0	0	0	0	0	2/7	5/6	3/7	0	0
*P_j_*(*a*_1_,*a*_3_)	5/9	0	0	0	0	1	0	0	1	0	0	0	0	0	0	0	0	0	3/7	5/6	0
*P_j_*(*a*_2_,*a*_1_)	0	0	2/7	5/9	5/6	0	3/7	5/6	0	5/6	1	1	0	1	5/6	1	0	0	0	0	5/6
*P_j_*(*a*_2_,*a*_3_)	0	0	0	0	0	1	3/7	5/6	1	5/6	5/6	0	0	1	3/7	1	0	0	0	5/6	3/7
*P_j_*(*a*_3_,*a*_1_)	0	5/12	1	1	5/6	0	0	0	0	0	5/12	1	1	1	5/12	0	5/18	0	0	0	5/12
*P_j_*(*a*_3_,*a*_2_)	5/6	3/7	5/6	5/9	0	0	0	0	0	0	0	1	1	0	0	0	5/9	5/6	0	0	0

**Table 4:** Priority index.

*π_j_*(*a*_1_,*a*_2_)	*π_j_*(*a*_1_,*a*_3_)	*π_j_*(*a*_2_,*a*_1_)	*π_j_*(*a*_2_,*a*_3_)	*π_j_*(*a*_3_,*a*_1_)	*π_j_*(*a*_3_,*a*_2_)
3/29	3/19	45/92	40/93	15/29	21/68

**Table 5:** Inflow and outflow for each scheme.

	*a*_1_	*a*_2_	*a*_3_
Outflow *φ^+^(a_i_)*	23/88	34/37	19/23
Inflow *φ*^−^(*a_i_*)	100/99	40/97	47/80
